# Supplementary material for: Computational design and cheminformatics profiling of omeprazole derivatives for enhanced proton pump inhibition of potassium-transporting ATPase alpha chain 1
Source: PLoS One. 2025 Jun 24;20(6):e0326655. doi: 10.1371/journal.pone.0326655 (PMC12186943; doi:10.1371/journal.pone.0326655)
Supplement: S1 File — S1 Table. Chemical structure and IUPAC name of the OMP and its analogues. S1 Fig. Optimized chemical structure of OMP and its analogues. S2 Fig. Molecular orbitals (HOMO and LUMO) and HUMO-LOMO energy gap of OMP and its analogues. S3 Fig. Free energies (Hartree) (a), dipole moments (Debye) (b), HOMO-LUMO gaps (c), and chemical softness (d) of OMP and its analogues. S2 Table. Energy (eV) of HOMO-LUMO, gap, hardness (η), softness (S), chemical potential (μ), electronegativity (χ), and electrophilicity (ω) of OMP, and its analogues. S4 Fig. Electrostatic potential map of OMP analogues. S5 Fig. FT-IR spectra of OMP analogues. S6 Fig. UV-vis spectra of OMP analogues. S3 Table. Vibrational frequencies of OMP analogues. S4 Table. UV-vis spectral data of OMP and its analogues. S7 Fig. Binding energy of OMP and its analogues with the targeted PTAAC1 protein. S5 Table. Binding affinity and nonbonding interactionss of remaining compounds with the PTAAC1 protein. S8 Fig. Superimposed view of the docked conformer with the targeted protein, non-bonding interactions, and the hydrogen bond surface of OMP and its analogues. S6 Table. Absorption, distribution, metabolism, and toxicological properties (ADMET) studies of OMP analogues. S7 Table. PASS predicted data of OMP analogues. (DOCX) [file pone.0326655.s001.docx]

**Computational design and cheminformatics profiling of omeprazole derivatives for enhanced proton pump inhibition of potassium-transporting ATPase alpha chain 1**

Mahmudul Hasan ^1, 2^, Md. Ifteker Hossain ^1, 2^, Noimul Hasan Siddiquee ^1^, Ezaz Ahmed ^1, 2^, Md Walid Hossain Talukder ^2, 3^, Md Rahamatolla ^2, 4^, Tasrin Nahar ^2, 5^, Popy Rani Paul ^2, 6^, Mahmudul Hassan Suhag ^7^, Monir Uzzaman ^2, 6^*

^1^ Faculty of Science, Department of Microbiology, Noakhali Science and Technology University, Noakhali 3814, Bangladesh.

^2^ Drug Design Division, Computer in Chemistry and Medicine Laboratory, Dhaka, Bangladesh.

^3^ Faculty of Science, Department of Applied Chemistry and Chemical Engineering, University of Chittagong, Chittagong, 4331, Bangladesh.

^4^ Faculty of Science, Department of Chemistry, University of Rajshahi, Rajshahi 6205, Bangladesh.

^5^ School of Physical Science, Department of Chemistry, Shahjalal University of Science and Technology, Sylhet 3114, Bangladesh.

^6^ Faculty of Science, Department of Chemistry, University of Chittagong, Chittagong, 4331, Bangladesh.

^7^ Faculty of Science and Engineering, Department of Chemistry, University of Barishal, Barishal 8254, Bangladesh.

*Correspondence:

Monir Uzzaman

Faculty of Science, Department of Chemistry

University of Chittagong, Chittagong 4331, Bangladesh.

Email: [monircu92@gmail.com](file:///C:\Users\User\AppData\Local\Microsoft\Windows\INetCache\IE\63UWJ1VO\monircu92@gmail.com)

| **Serial** | **Title** |
| --- | --- |
| Table Supplementary S1 | Chemical structure and IUPAC name of the OMP and its analogues |
| Figure Supplementary S1 | Optimized chemical structure of OMP and its analogues |
| Figure Supplementary S2 | Molecular orbitals (HOMO and LUMO) and HUMO-LOMO energy gap of OMP and its analogues |
| Figure Supplementary S3 | **Free energies** (Hartree) **(a), dipole moments** (Debye) **(b), chemical softness (c), and HOMO-LUMO gaps (d) of OMP and its analogues** |
| Table Supplementary S2 | Energy (eV) of HOMO-LUMO, gap, hardness (η), softness (S), chemical potential (μ), electronegativity (χ), and electrophilicity (ω) of OMP, and its analogues |
| Figure Supplementary S4 | Electrostatic potential map of OMP analogues |
| Figure Supplementary S5 | FT-IR spectra of OMP analogues |
| Figure Supplementary S6 | UV-vis spectra of OMP analogues |
| Table Supplementary S3 | Vibrational frequencies of OMP analogues |
| Table Supplementary S4 | UV-vis spectral data of OMP and its analogues |
| Figure Supplementary S7 | Binding energy of OMP and its analogues with the targeted PTAAC1 protein |
| Table Supplementary S5 | Binding affinity and nonbonding interactions of remaining compounds with the PTAAC1 protein |
| Figure Supplementary S8 | Superimposed view of the docked conformer with the targeted protein, non-bonding interactions, and the hydrogen bond surface of OMP and its analogues |
| Table Supplementary S6 | Absorption, distribution, metabolism, and toxicological properties (ADMET) studies of OMP analogues |
| Table Supplementary S7 | PASS predicted data of OMP analogues |

**Table Supplementary S1. Chemical structure and IUPAC name of the OMP and its analogues**

| Name | Structure |
| --- | --- |
| OMP1 |  |
| OMP2 |  |
| OMP3 |  |
| OMP4 |  |
| OMP5 |  |
| OMP6 |  |
| OMP7 |  |
| OMP8 |  |
| OMP9 |  |
| OMP10 |  |
| OMP11 |  |
| OMP12 |  |
| OMP13 |  |
| OMP14 |  |
| OMP15 |  |
| OMP16 |  |
| OMP17 |  |
| OMP18 |  |
| OMP19 |  |
| OMP20 |  |
| OMP21 |  |
| OMP22 |  |

**Figure Supplementary S1. Optimized chemical structure of OMP and its analogues**

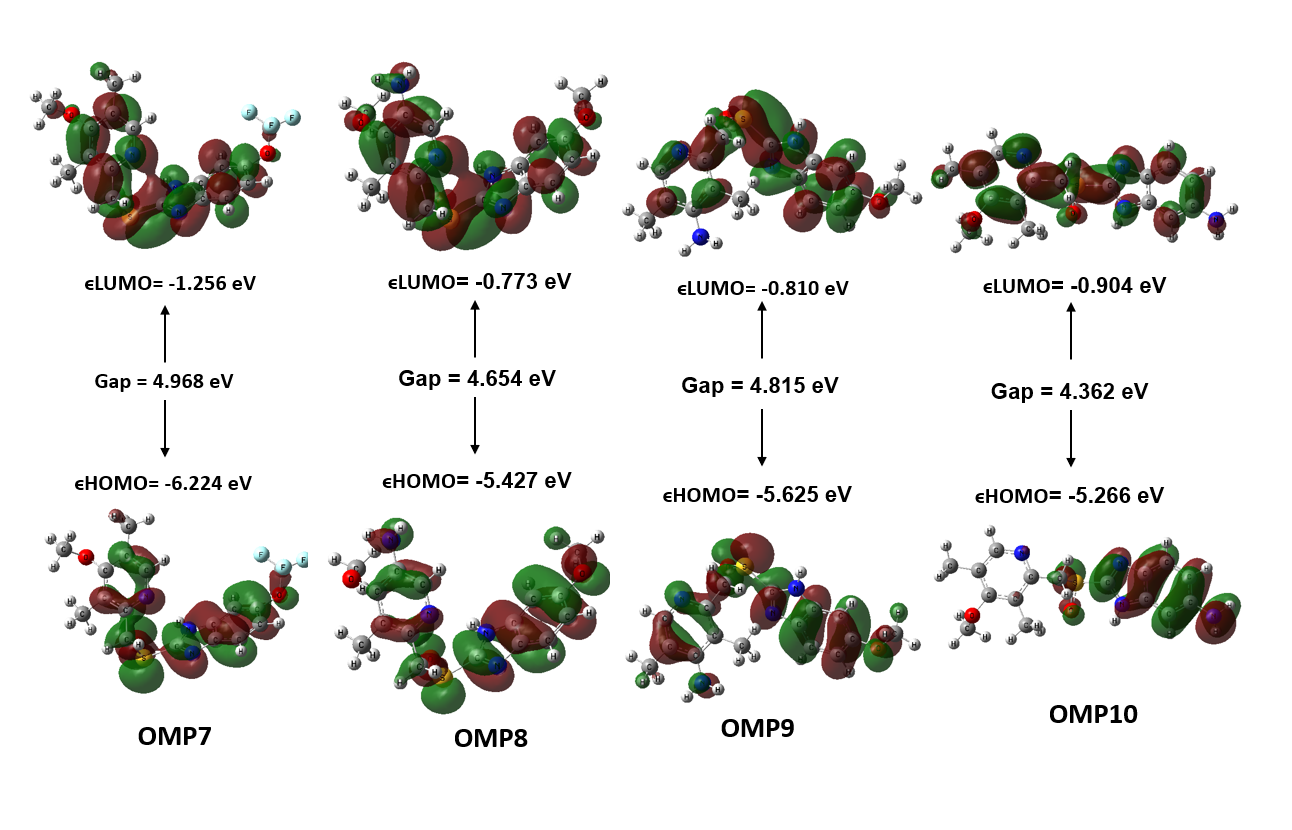


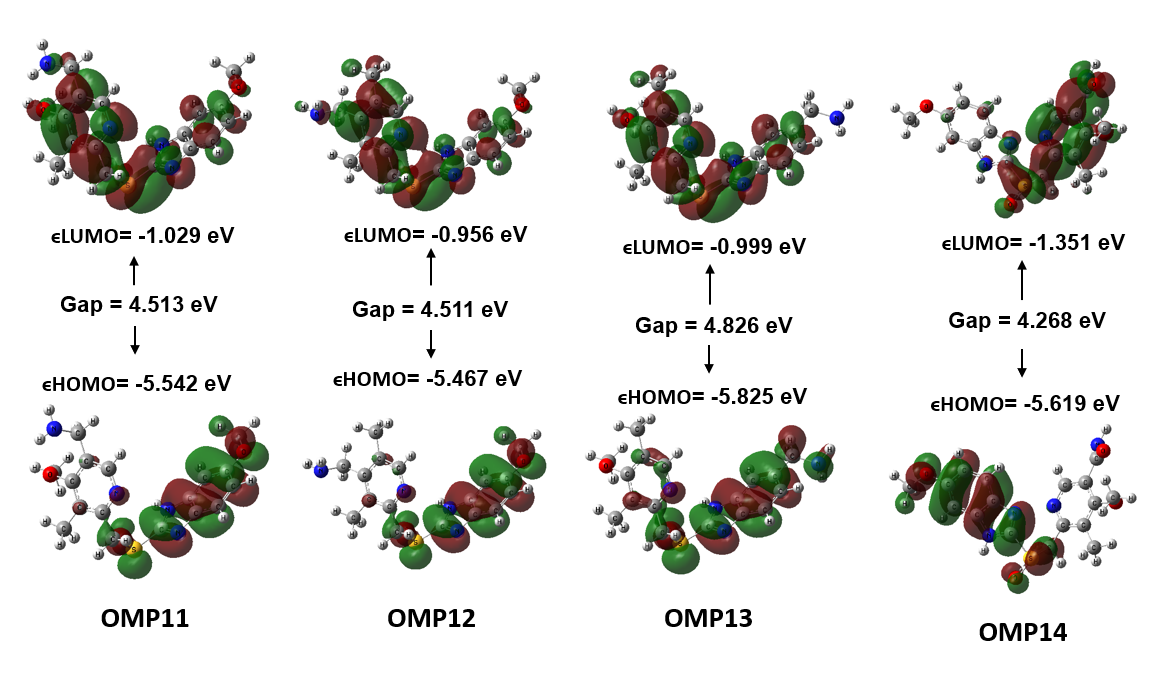


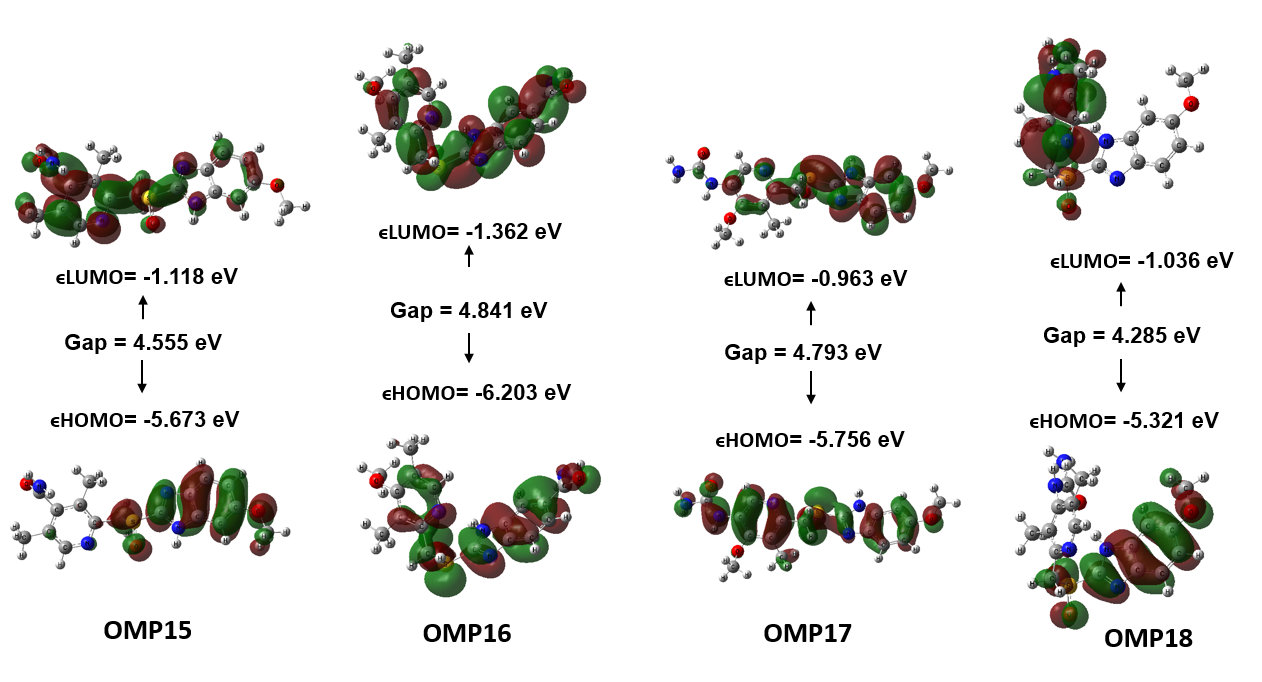


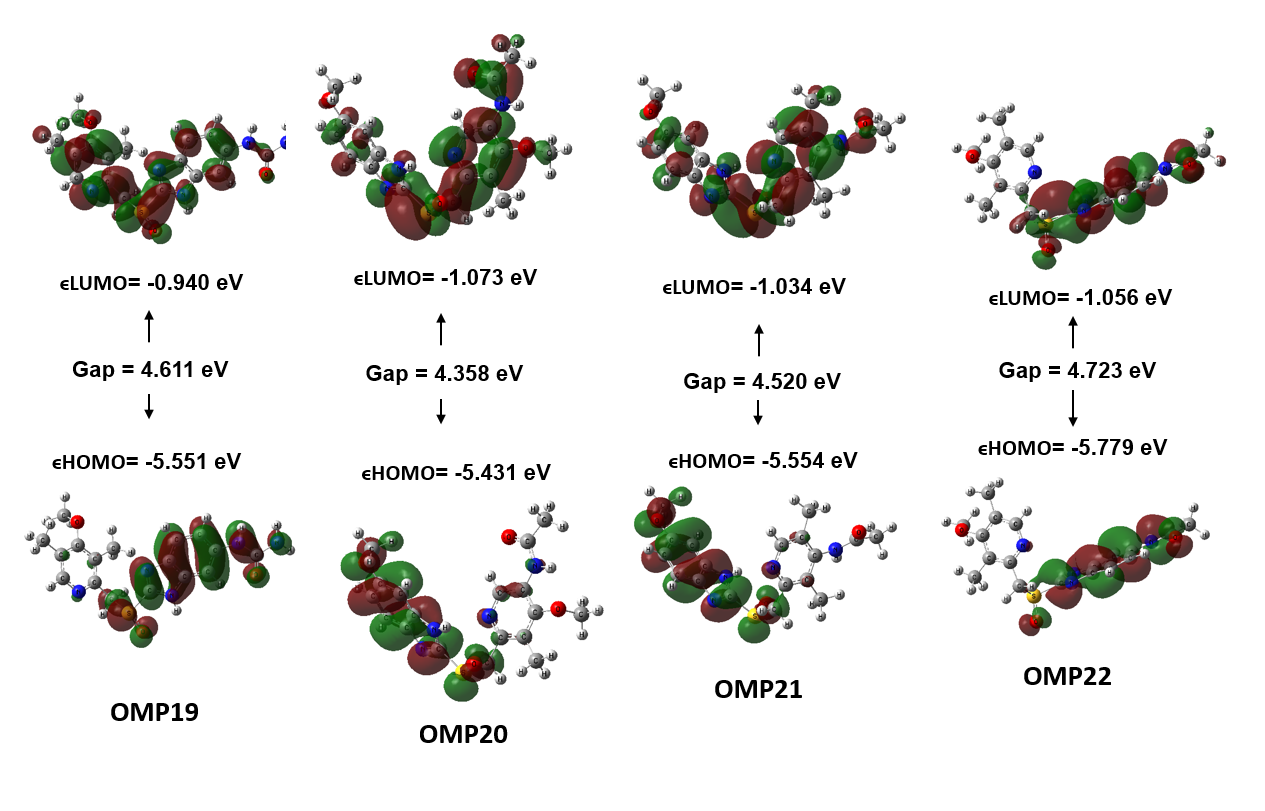


**Figure Supplementary S2. Molecular orbitals (HOMO and LUMO) and HUMO-LOMO energy gap of OMP and its analogues**

**Figure Supplementary S3. Free energies (Hartree) (a), dipole moments (Debye)** **(b), HOMO-LUMO gaps (c), and chemical softness (d) of OMP and its analogues**

**Table Supplementary S2. Energy (eV) of HOMO-LUMO, gap, hardness (η), softness (S), chemical potential (μ), electronegativity (χ), and electrophilicity (ω) of OMP, and its analogues**

| Name | ϵHOMO | ϵLUMO | Gap | η | S | μ | χ | ω |
| --- | --- | --- | --- | --- | --- | --- | --- | --- |
| OMP3 | -5.742 | -1.317 | 4.425 | 2.213 | 0.452 | -3.530 | 3.530 | 2.815 |
| OMP4 | -6.352 | -1.332 | 5.020 | 2.510 | 0.398 | -3.842 | 3.842 | 2.940 |
| OMP6 | -5.700 | -1.196 | 4.503 | 2.252 | 0.444 | -3.448 | 3.448 | 2.640 |
| OMP7 | -6.224 | -1.256 | 4.967 | 2.484 | 0.403 | -3.740 | 3.740 | 2.816 |
| OMP9 | -5.625 | -0.810 | 4.815 | 2.407 | 0.415 | -3.217 | 3.217 | 2.150 |
| OMP10 | -5.266 | -0.904 | 4.362 | 2.181 | 0.459 | -3.085 | 3.085 | 2.182 |
| OMP12 | -5.467 | -0.956 | 4.510 | 2.255 | 0.443 | -3.212 | 3.212 | 2.287 |
| OMP13 | -5.825 | -0.999 | 4.826 | 2.413 | 0.414 | -3.412 | 3.412 | 2.412 |
| OMP15 | -5.673 | -1.118 | 4.555 | 2.277 | 0.439 | -3.395 | 3.395 | 2.531 |
| OMP16 | -6.203 | -1.362 | 4.841 | 2.42 | 0.413 | -3.783 | 3.783 | 2.956 |
| OMP18 | -5.321 | -1.036 | 4.285 | 2.142 | 0.467 | -3.178 | 3.178 | 2.358 |
| OMP19 | -5.551 | -0.94 | 4.612 | 2.306 | 0.434 | -3.246 | 3.246 | 2.284 |
| OMP21 | -5.554 | -1.034 | 4.520 | 2.260 | 0.442 | -3.294 | 3.294 | 2.401 |
| OMP22 | -5.779 | -1.056 | 4.723 | 2.362 | 0.423 | -3.417 | 3.417 | 2.472 |

 **Figure Supplementary S4. Electrostatic potential map of OMP analogues**

**Figure Supplementary S5. FT-IR spectra of OMP analogues**

**Figure Supplementary S6. UV-vis spectra of OMP analogues.**

**Table Supplementary S3. Vibrational frequencies of OMP analogues**

| Name | Assignment | Vibrational frequencies (cm^-1^) | Corrected vibrational frequencies (cm^-1^) |
| --- | --- | --- | --- |
| OMP | νN-H^a^ stretch | 3631 | 3503 |
|  | νS=O stretch | 1016 | 980 |
|  | νC=N stretch | 1671 | 1612 |
|  | νC-H^a^ stretch | 3227 | 3113 |
|  | νC-H stretch | 3046 | 2939 |
| OMP1 | νN-H^a^ stretch | 3625 | 3497 |
|  | νS=O stretch | 1014 | 978 |
|  | νC=N stretch | 1672 | 1613 |
|  | νC-H^a^ stretch | 3200 | 3087 |
|  | νC-H stretch | 3046 | 2939 |
| OMP2 | νN-H^a^ stretch | 3668 | 3539 |
|  | νS=O stretch | 1051 | 1014 |
|  | νC=N stretch | 1682 | 1623 |
|  | νC-H^a^ stretch | 3223 | 3110 |
|  | νC-H stretch | 3013 | 2907 |
| OMP3 | νN-H^a^ stretch | 3668 | 3539 |
|  | νS=O stretch | 1083 | 1045 |
|  | νC=N stretch | 1682 | 1623 |
|  | νC-H^a^ stretch | 3229 | 3115 |
|  | νC-H stretch | 3013 | 2907 |
| OMP4 | νN-H^a^ stretch | 3630 | 3502 |
|  | νS=O stretch | 1075 | 1037 |
|  | νC=N stretch | 1678 | 1619 |
|  | νC-H^a^ stretch | 3224 | 3111 |
|  | νC-H stretch | 3025 | 2919 |
| OMP5 | νN-H^a^ stretch | 3637 | 3509 |
|  | νS=O stretch | 1038 | 1001 |
|  | νC=N stretch | 1682 | 1623 |
|  | νC-F stretch | 1212 | 1169 |
|  | νC-H^a^ stretch | 3207 | 3094 |
|  | νC-H stretch | 3012 | 2906 |
| OMP6 | νN-H^a^ stretch | 3624 | 3496 |
|  | νS=O stretch | 1042 | 1005 |
|  | νC=N stretch | 1680 | 1621 |
|  | νC-F stretch | 1207 | 1165 |
|  | νC-H^a^ stretch | 3226 | 3112 |
|  | νC-H stretch | 3013 | 2907 |
| OMP7 | νN-H^a^ stretch | 3629 | 3501 |
|  | νS=O stretch | 1042 | 1005 |
|  | νC=N stretch | 1679 | 1620 |
|  | νC-F stretch | 1213 | 1170 |
|  | νC-H^a^ stretch | 3157 | 3046 |
|  | νC-H stretch | 3022 | 2916 |
| OMP8 | νN-H^a^ stretch | 3637 | 3509 |
|  | νN-H stretch | 3558 | 3433 |
|  | νS=O stretch | 1049 | 1012 |
|  | νC=N stretch | 1681 | 1622 |
|  | νC-H^a^ stretch | 3230 | 3116 |
|  | νC-H stretch | 3010 | 2904 |
| OMP9 | νN-H^a^ stretch | 3662 | 3533 |
|  | νN-H stretch | 3606 | 3479 |
|  | νS=O stretch | 1112 | 1073 |
|  | νC=N stretch | 1680 | 1621 |
|  | νC-H^a^ stretch | 3222 | 3109 |
|  | νC-H stretch | 3012 | 2906 |
| OMP10 | νN-H^a^ stretch | 3635 | 3507 |
|  | νN-H stretch | 3568 | 3442 |
|  | νS=O stretch | 1032 | 996 |
|  | νC=N stretch | 1690 | 1631 |
|  | νC-H^a^ stretch | 3214 | 3101 |
|  | νC-H stretch | 3025 | 2919 |
| OMP11 | νN-H^a^ stretch | 3641 | 3513 |
|  | νS=O stretch | 1039 | 1002 |
|  | νC=N stretch | 1682 | 1623 |
|  | νC-H^a^ stretch | 3221 | 3108 |
|  | νC-H stretch | 3011 | 2905 |
| OMP12 | νN-H^a^ stretch | 3636 | 3508 |
|  | νS=O stretch | 1038 | 1001 |
|  | νC=N stretch | 1681 | 1622 |
|  | νC-H^a^ stretch | 3221 | 3108 |
|  | νC-H stretch | 3040 | 2933 |
| OMP13 | νN-H^a^ stretch | 3639 | 3511 |
|  | νS=O stretch | 1030 | 994 |
|  | νC=N stretch | 1681 | 1622 |
|  | νC-H^a^ stretch | 3219 | 3106 |
|  | νC-H stretch | 3025 | 2919 |
| OMP14 | νN-H^a^ stretch | 3633 | 3505 |
|  | νN-H stretch | 3594 | 3467 |
|  | νS=O stretch | 1039 | 1002 |
|  | νC=N stretch | 1678 | 1619 |
|  | νC=O stretch | 1786 | 1723 |
|  | νC-H^a^ stretch | 3222 | 3109 |
|  | νC-H stretch | 3049 | 2942 |
| OMP15 | νN-H^a^ stretch | 3629 | 3501 |
|  | νN-H stretch | 3595 | 3468 |
|  | νS=O stretch | 1054 | 1017 |
|  | νC=N stretch | 1681 | 1622 |
|  | νC=O stretch | 1791 | 1728 |
|  | νC-H^a^ stretch | 3222 | 3109 |
|  | νC-H stretch | 3013 | 2907 |
| OMP16 | νN-H^a^ stretch | 3632 | 3504 |
|  | νS=O stretch | 1039 | 1002 |
|  | νC=N stretch | 1632 | 1575 |
|  | νC=O stretch | 1784 | 1721 |
|  | νC-H^a^ stretch | 3203 | 3090 |
|  | νC-H stretch | 3025 | 2919 |
| OMP17 | νN-H^a^ stretch | 3663 | 3534 |
|  | νN-H stretch | 3578 | 3452 |
|  | νS=O stretch | 1030 | 994 |
|  | νC=N stretch | 1682 | 1623 |
|  | νC=O stretch | 1813 | 1749 |
|  | νC-H^a^ stretch | 3209 | 3096 |
|  | νC-H stretch | 3033 | 2926 |
| OMP18 | νN-H^a^ stretch | 3504 | 3381 |
|  | νN-H stretch | 3713 | 3582 |
|  | νS=O stretch | 1102 | 1063 |
|  | νC=N stretch | 1681 | 1622 |
|  | νC=O stretch | 1787 | 1724 |
|  | νC-H^a^ stretch | 3221 | 3108 |
|  | νC-H stretch | 3007 | 2901 |
| OMP19 | νN-H^a^ stretch | 3621 | 3494 |
|  | νN-H stretch | 3575 | 3449 |
|  | νS=O stretch | 1037 | 1000 |
|  | νC=N stretch | 1682 | 1623 |
|  | νC=O stretch | 1808 | 1744 |
|  | νC-H^a^ stretch | 3265 | 3150 |
|  | νC-H stretch | 3042 | 2935 |
| OMP20 | νN-H^a^ stretch | 3640 | 3512 |
|  | νN-H stretch | 3612 | 3485 |
|  | νS=O stretch | 1037 | 1000 |
|  | νC=N stretch | 1682 | 1623 |
|  | νC=O stretch | 1791 | 1728 |
|  | νC-H^a^ stretch | 3207 | 3094 |
|  | νC-H stretch | 3038 | 2931 |
| OMP21 | νN-H^a^ stretch | 3641 | 3513 |
|  | νN-H stretch | 3619 | 3492 |
|  | νS=O stretch | 1036 | 1000 |
|  | νC=N stretch | 1682 | 1623 |
|  | νC=O stretch | 1804 | 1740 |
|  | νC-H^a^ stretch | 3231 | 3117 |
|  | νC-H stretch | 3011 | 2905 |
| OMP22 | νN-H^a^ stretch | 3668 | 3539 |
|  | νN-H stretch | 3637 | 3509 |
|  | νS=O stretch | 1036 | 1000 |
|  | νC=N stretch | 1687 | 1628 |
|  | νC=O stretch | 1790 | 1727 |
|  | νC-H^a^ stretch | 3269 | 3154 |
|  | νC-H stretch | 3023 | 2917 |

Here, a=aromatic

**Table Supplementary S4. UV-vis spectral data of OMP and its analogues**

| **Name** | **Excited State** | **Wavelength (nm)** | **Excitation Energy (eV)** | **Configurations Composition** | **Oscillator Strength** |
| --- | --- | --- | --- | --- | --- |
| OMP | S_0_→ S_1_ | 680.250 | 1.823 | 0.700(H → L) | 0.043 |
|  | S_0_→ S_2_ | 377.260 | 3.287 | 0.599 (H → L+1), -0.358(H → L+2) | 0.006 |
| OMP1 | S_0_→ S_1_ | 735.070 | 1.687 | 0.701(H → L) | 0.009 |
|  | S_0_→ S_2_ | 396.770 | 3.125 | 0.701(H-1 → L) | 0.067 |
| OMP2 | S_0_→ S_1_ | 631.790 | 1.962 | 0.698(H → L) | 0.051 |
|  | S_0_→ S_2_ | 426.590 | 2.906 | 0.691(H → L+1) | 0.018 |
| OMP3 | S_0_→ S_1_ | 953.120 | 1.301 | 0.703(H → L) | 0.009 |
|  | S_0_→ S_2_ | 539.490 | 2.298 | 0.703(H → L+1) | 0.013 |
| OMP4 | S_0_→ S_1_ | 731.520 | 1.695 | 0.705(H → L) | 0.041 |
|  | S_0_→ S_2_ | 376.900 | 3.289 | 0.702(H-1→ L) | 0.003 |
| OMP5 | S_0_→ S_1_ | 701.820 | 1.766 | 0.700(H → L) | 0.035 |
|  | S_0_→ S_2_ | 403.360 | 3.073 | 0.103(H-1 → L), 0.657(H → L+1), 0.223(H → L+2) | 0.023 |
| OMP6 | S_0_→ S_1_ | 1008.660 | 1.229 | 0.704 (H → L) | 0.003 |
|  | S_0_→ S_2_ | 496.610 | 2.496 | 0.689(H → L+1), 0.145(H → L+2) | 0.004 |
| OMP7 | S_0_→ S_1_ | 697.650 | 1.777 | 0.702(H → L) | 0.045 |
|  | S_0_→ S_2_ | 366.690 | 3.381 | 0.685(H → L+1), 0.100(H → L+2) | 0.005 |
| OMP8 | S_0_→ S_1_ | 695.100 | 1.783 | 0.701 (H → L) | 0.041 |
|  | S_0_→ S_2_ | 409.020 | 3.031 | 0.703(H-1 → L) | 0.008 |
| OMP9 | S_0_→ S_1_ | 858.110 | 1.445 | 0.702(H → L) | 0.005 |
|  | S_0_→ S_2_ | 416.190 | 2.979 | 0.704(H-1 → L) | 0.000 |
| OMP10 | S_0_→ S_1_ | 802.160 | 1.546 | 0.702(H → L) | 0.008 |
|  | S_0_→ S_2_ | 383.140 | 3.236 | -0.122(H-2 → L), 0.673(H-1 → L) | 0.250 |
| OMP11 | S_0_→ S_1_ | 667.730 | 1.857 | 0.701(H → L) | 0.046 |
|  | S_0_→ S_2_ | 382.290 | 3.243 | 0.688(H → L+1) | 0.011 |
| OMP12 | S_0_→ S_1_ | 678.890 | 1.826 | 0.701(H → L) | 0.044 |
|  | S_0_→ S_2_ | 411.250 | 3.015 | 0.701(H → L+1) | 0.009 |
| OMP13 | S_0_→ S_1_ | 700.740 | 1.769 | 0.704(H → L) | 0.043 |
|  | S_0_→ S_2_ | 367.930 | 3.370 | 0.657(H → L+1), 0.225(H → L+2) | 0.005 |
| OMP14 | S_0_→ S_1_ | 1052.390 | 1.178 | 0.705(H → L) | 0.000 |
|  | S_0_→ S_2_ | 547.060 | 2.266 | 0.704(H → L+1) | 0.000 |
| OMP15 | S_0_→ S_1_ | 907.230 | 1.367 | 0.705(H → L) | 0.000 |
|  | S_0_→ S_2_ | 461.980 | 2.684 | 0.705(H → L+1) | 0.033 |
| OMP16 | S_0_→ S_1_ | 747.050 | 1.659 | 0.704 (H → L) | 0.046 |
|  | S_0_→ S_2_ | 382.270 | 3.243 | 0.186(H-2 → L), -0.137(H-1 → L), 0.655(H → L+1) | 0.003 |
| OMP17 | S_0_→ S_1_ | 820.080 | 1.512 | 0.702(H → L) | 0.008 |
|  | S_0_→ S_2_ | 379.360 | 3.268 | 0.326(H-3 → L), 0.322(H-2 → L), 0.522(H-1 → L) | 0.059 |
| OMP18 | S_0_→ S_1_ | 880.690 | 1.408 | 0.704 (H → L) | 0.002 |
|  | S_0_→ S_2_ | 474.200 | 2.615 | 0.700(H → L+1) | 0.000 |
| OMP19 | S_0_→ S_1_ | 1130.760 | 1.097 | 0.704(H → L) | 0.003 |
|  | S_0_→ S_2_ | 414.680 | 2.990 | 0.700(H-1 → L) | 0.006 |
| OMP20 | S_0_→ S_1_ | 715.850 | 1.732 | 0.702 (H → L) | 0.037 |
|  | S_0_→ S_2_ | 385.110 | 3.220 | 0.105(H-1 → L), 0.656(H-→ L+1), 0.220(H → L+2) | 0.022 |
| OMP21 | S_0_→ S_1_ | 762.640 | 1.626 | 0.702 (H → L) | 0.027 |
|  | S_0_→ S_2_ | 416.980 | 2.973 | 0.697(H → L+1) | 0.006 |
| OMP22 | S_0_→ S_1_ | 1124.860 | 1.102 | 0.704(H → L) | 0.000 |
|  | S_0_→ S_2_ | 400.020 | 3.099 | 0.104(H-7 → L), 0.190(H-3 → L),  0.630(H-1 → L), -0.182(H→ L+1) | 0.027 |

**Figure Supplementary S7. Binding energy of OMP and its analogues with the targeted protein**

**Table Supplementary S5. Binding affinity and nonbonding interactions of remaining compounds with the PTAAC1 protein**

| Name | Binding affinity (kcal/ mol) | Residues in contact | Interaction type | Distance (Å) |
| --- | --- | --- | --- | --- |
|  |  |  |  |  |
| OMP | -7 | ASN991 | H | 2.40238 |
|  |  | TYR801 | H | 2.96557 |
|  |  | ASP139 | H | 2.53565 |
|  |  | ALA337 | A | 4.01548 |
|  |  | VAL333 | A | 4.77205 |
|  |  | LEU923 | A | 5.33114 |
|  |  | TYR801 | PA | 5.31099 |
|  |  | PHE919 | PA | 4.84499 |
| OMP1 | -6.9 | LYS784 | H | 2.18179 |
|  |  | ARG951 | H | 2.26645 |
|  |  | ARG951 | H | 2.23142 |
|  |  | ILE842 | C | 3.72052 |
|  |  | TYR1034 | PPTS | 4.79512 |
|  |  | PRO859 | A | 4.11136 |
|  |  | LEU845 | A | 4.05241 |
| OMP2 | -7.6 | THR377 | H | 1.9841 |
|  |  | ASP740 | X | 2.57494 |
|  |  | ASP740 | X | 3.23782 |
|  |  | ILE741 | X | 2.54273 |
|  |  | ILE741 | X | 3.65494 |
|  |  | ASP758 | X | 3.57147 |
|  |  | THR377 | PS | 3.66624 |
|  |  | THR377 | PS | 3.52367 |
|  |  | ALA724 | PS | 3.02564 |
|  |  | VAL723; ALA724 | APS | 3.49734 |
|  |  | ALA724 | A | 2.97312 |
|  |  | ALA724 | A | 4.25842 |
|  |  | LEU378 | A | 3.6927 |
|  |  | ILE741 | A | 4.8048 |
|  |  | ILE741 | A | 3.66582 |
|  |  | PRO294 | PA | 4.25397 |
|  |  | LEU378 | PA | 5.21406 |
|  |  | ALA724 | A | 5.42028 |
| OMP3 | -7.9 | LYS784 | H | 2.03935 |
|  |  | ARG951 | H | 2.27993 |
|  |  | ASP853 | H | 2.68796 |
|  |  | ARG777 | X | 3.05064 |
|  |  | TYR1034 | PSu | 5.89964 |
|  |  | ARG848 | A | 3.45658 |
|  |  | ARG777 | A | 4.11721 |
|  |  | ARG848 | A | 4.30438 |
|  |  | ARG777 | A | 3.89063 |
|  |  | ILE842 | A | 4.49137 |
|  |  | LEU845 | A | 5.08436 |
|  |  | PHE780 | PA | 4.41439 |
|  |  | PHE780 | PA | 4.888 |
| OMP4 | -7.7 | THR377 | H | 1.86063 |
|  |  | VAL723 | A | 2.53367 |
|  |  | ALA724 | H | 2.6041 |
|  |  | VAL383 | A | 2.70677 |
|  |  | ALA721 | C | 2.76495 |
|  |  | ILE723 | C | 2.92456 |
|  |  | ASP740 | X | 3.30988 |
|  |  | ASP740 | X | 3.47536 |
|  |  | ALA724 | X | 3.53266 |
|  |  | ALA724 | PA | 3.59959 |
|  |  | ALA724 | A | 3.6595 |
|  |  | LEU378 | A | 3.70836 |
|  |  | VAL743 | Pa | 3.7175 |
|  |  | LEU378 | A | 3.92507 |
|  |  | ILE384 | PA | 4.30312 |
|  |  | PRO294 | A | 4.38269 |
|  |  | PRO294 | PA | 4.40661 |
|  |  | ILE722 | A | 4.61806 |
|  |  | ILE741 | PA | 5.18402 |
|  |  | ALA724 | A | 5.42028 |
| OMP5 | -7.5 | LYS784 | H | 2.14745 |
|  |  | ARG848 | H | 2.72845 |
|  |  | ARG848 | H | 2.88143 |
|  |  | ARG951 | H | 2.85313 |
|  |  | ARG951 | H | 2.06114 |
|  |  | ASP781 | H | 3.01451 |
|  |  | ARG777 | C | 2.94342 |
|  |  | GLY379 | X | 2.98125 |
|  |  | GLY776 | X | 3.62774 |
|  |  | ARG848 | A | 4.79699 |
|  |  | ARG777 | A | 3.7711 |
|  |  | ARG848 | A | 3.94519 |
|  |  | PHE780 | PA | 4.57026 |
| OMP6 | -7.5 | THR230 | H | 2.72845 |
|  |  | GLU234 | H | 2.4134 |
|  |  | ASP630 | H | 3.07494 |
|  |  | GLN236 | X | 3.4773 |
|  |  | GLN236 | X | 3.48787 |
|  |  | GLU232 | Pa | 3.17727 |
|  |  | ALA462 | A | 3.86626 |
|  |  | ARG703 | A | 4.63275 |
|  |  | MET397 | A | 4.87602 |
|  |  | MET397 | PA | 5.33999 |
| OMP7 | -7.6 | CYS815 | H | 2.51075 |
|  |  | ILE816 | H | 2.36881 |
|  |  | ASN140 | H | 2.71905 |
|  |  | THR136 | C | 3.62412 |
|  |  | ASP139 | C | 3.62586 |
|  |  | THR136 | X | 3.34809 |
|  |  | HIS904 | X | 3.49557 |
|  |  | LEU143 | A | 3.76446 |
|  |  | CYS815 | A | 4.51512 |
|  |  | ILE816 | A | 4.45713 |
|  |  | LEU811 | A | 4.78868 |
|  |  | CYS815 | A | 4.39511 |
|  |  | LEU923 | A | 4.94912 |
|  |  | HIS904 | PA | 4.95947 |
|  |  | PHE919 | PA | 5.2797 |
| OMP8 | -7.1 | THR377 | H | 2.26098 |
|  |  | VAL723 | H | 2.72026 |
|  |  | ASP740 | Pa | 3.6384 |
|  |  | LEU378 | PS | 2.28463 |
|  |  | ALA724 | A | 3.0167 |
|  |  | LEU378 | A | 4.10316 |
|  |  | VAL743 | A | 5.03577 |
|  |  | LEU378 | A | 5.16818 |
|  |  | ILE384 | A | 4.02147 |
|  |  | ILE722 | PA | 4.5345 |
|  |  | ILE741 | PA | 5.09244 |
|  |  | ALA724 | PA | 3.88878 |
| OMP9 | -7.1 | ASP781 | H | 2.74118 |
|  |  | SER380 | C | 3.58425 |
|  |  | ASP781 | Pa | 3.96915 |
|  |  | PHE780 | PPS | 5.32533 |
|  |  | ARG952 | A | 4.04219 |
|  |  | LEU953 | A | 4.38096 |
|  |  | LEU845 | A | 4.78722 |
|  |  | ARG777 | A | 3.99037 |
|  |  | ARG848 | A | 3.95879 |
|  |  | PHE780 | PA | 4.35751 |
|  |  | ARG777 | PA | 5.11133 |
|  |  | ARG848 | PA | 4.64078 |
| OMP10 | -7.4 | ALA724 | H | 2.49113 |
|  |  | ASP740 | H | 2.74262 |
|  |  | ALA374 | C | 2.66098 |
|  |  | ASP740 | Pa | 4.0512 |
|  |  | ASP740 | Pa | 4.28587 |
|  |  | ALA724 | A | 3.43959 |
|  |  | ALA724 | A | 3.61121 |
|  |  | ALA724 | A | 3.58651 |
|  |  | ILE741 | A | 4.31325 |
|  |  | LEU378 | A | 3.24223 |
|  |  | ILE741 | A | 5.2956 |
|  |  | VAL773 | A | 5.07866 |
|  |  | PRO294 | PA | 3.7545 |
|  |  | ILE741 | PA | 5.28328 |
|  |  | ALA724 | PA | 3.00851 |
| OMP11 | -6.9 | ILE722 | H | 2.89238 |
|  |  | VAL723 | H | 2.54231 |
|  |  | SER380 | H | 1.89417 |
|  |  | ALA739 | C | 2.77005 |
|  |  | ASP758 | Pa | 4.77282 |
|  |  | ILE722 | A | 4.09587 |
|  |  | PRO294 | A | 5.36282 |
|  |  | ALA374 | PA | 5.43274 |
|  |  | LEU378 | PA | 5.48655 |
|  |  | ALA724 | PA | 4.73559 |
|  |  | PRO294 | PA | 5.46251 |
|  |  | ALA374 | PA | 4.63261 |
| OMP12 | -7.2 | ARG618 | H | 2.46536 |
|  |  | PRO847 | H | 2.69255 |
|  |  | PRO847 | H | 2.7883 |
|  |  | GLY621 | H | 2.18386 |
|  |  | VAL624 | C | 3.60978 |
|  |  | PRO847 | A | 3.89288 |
|  |  | ARG623 | A | 4.50765 |
|  |  | ARG618 | PA | 5.33972 |
|  |  | ARG623 | PA | 3.97141 |
| OMP13 | -6.9 | VAL723 | H | 2.73993 |
|  |  | ASP740 | Pa | 3.85309 |
|  |  | LEU378 | PS | 3.17731 |
|  |  | ALA724 | A | 3.12352 |
|  |  | ALA724 | A | 4.05402 |
|  |  | LEU378 | A | 4.10434 |
|  |  | VAL743 | A | 4.85571 |
|  |  | LEU378 | A | 4.99581 |
|  |  | LEU378 | A | 5.11677 |
|  |  | ILE384 | A | 3.74072 |
|  |  | ILE741 | PA | 5.08058 |
|  |  | ALA724 | PA | 3.67373 |
| OMP14 | -7.5 | ILE741 | HB | 2.58792 |
|  |  | Unknown | HB | 2.61305 |
|  |  | SER380 | HB | 2.67435 |
|  |  | ALA724 | HP | 4.11463 |
|  |  | LEU378 | HP | 4.11642 |
|  |  | VAL743 | HP | 5.25064 |
|  |  | LEU378 | HP | 4.99469 |
|  |  | ILE741 | HP | 4.22707 |
|  |  | VAL743 | HP | 5.44997 |
|  |  | PRO294 | HP | 3.84527 |
|  |  | PRO850 | HP | 4.19964 |
|  |  | PRO294 | HP | 3.78927 |
|  |  | ILE722 | HP | 4.26493 |
|  |  | ILE741 | HP | 5.47896 |
|  |  | LEU378 | HP | 4.18129 |
|  |  | ALA724 | HP | 3.84543 |
| OMP15 | -7.5 | SER380 | C | 3.4346 |
|  |  | ASP740 | C | 2.48213 |
|  |  | ASP740 | Pa | 4.72389 |
|  |  | ASP740 | Pa | 4.60826 |
|  |  | ALA724 | A | 4.36431 |
|  |  | LEU378 | A | 4.91628 |
|  |  | ILE741 | A | 4.63297 |
|  |  | LEU378 | A | 3.90791 |
|  |  | ILE741 | A | 3.67467 |
|  |  | MET759 | A | 4.48536 |
|  |  | ILE769 | A | 5.2811 |
|  |  | PRO294 | A | 4.58904 |
|  |  | ILE297 | A | 5.09005 |
| OMP16 | -7.5 | ARG330 | H | 2.78247 |
|  |  | GLN926 | H | 2.74519 |
|  |  | TYR930 | H | 2.26343 |
|  |  | ASP139 | H | 2.07805 |
|  |  | ARG330 | Pc | 4.84984 |
|  |  | TYR927 | PSu | 5.2049 |
|  |  | TYR930 | PSu | 5.74034 |
| OMP17 | -7.8 | ARG848 | H | 2.25997 |
|  |  | ASP781 | H | 2.93852 |
|  |  | SER380 | H | 2.18075 |
|  |  | ASP781 | Pa | 4.03505 |
|  |  | LEU845 | A | 3.87126 |
|  |  | ARG952 | A | 4.01963 |
| OMP18 | -7.6 | THR377 | H | 1.93182 |
|  |  | ALA374 | H | 2.91846 |
|  |  | THR377 | C | 3.48251 |
|  |  | SER380 | C | 3.56871 |
|  |  | THR377 | PS | 3.80267 |
|  |  | ALA724 | A | 2.72491 |
|  |  | ALA739 | A | 4.18385 |
|  |  | LEU378 | A | 3.54091 |
|  |  | ILE741 | A | 4.32905 |
| OMP19 | -8.3 | LEU378 | C | 2.50852 |
|  |  | SER380 | C | 2.57251 |
|  |  | ILE722 | C | 2.25577 |
|  |  | ILE722 | C | 2.1259 |
|  |  | ILE722 | PS | 3.60635 |
|  |  | ILE722 | A | 3.70575 |
|  |  | PRO294 | PA | 5.27908 |
|  |  | LEU378 | PA | 4.55256 |
|  |  | ILE741 | PA | 5.26048 |
| OMP20 | -7.2 | VAL723 | H | 2.83487 |
|  |  | THR293 | C | 3.03032 |
|  |  | ALA724 | A | O2.9419 |
|  |  | LEU378 | A | 5.37431 |
|  |  | ILE384 | A | 4.97366 |
|  |  | ILE722 | A | 3.49631 |
|  |  | VAL723 | A | 4.76406 |
|  |  | PRO294 | A | 4.55781 |
|  |  | ALA374 | PA | 4.99634 |
|  |  | ALA724 | PA | 5.33424 |
|  |  | ALA724 | PA | 4.64877 |
|  |  | ILE741 | PA | 5.17289 |
|  |  | PRO294 | PA | 5.11439 |
|  |  | ALA374 | PA | 5.04441 |
| OMP21 | -8.1 | THR381 | H | 2.71146 |
|  |  | ASP740 | C | 2.1039 |
|  |  | GLU376 | C | 2.25455 |
|  |  | ALA374 | A | 3.63098 |
|  |  | LEU378 | A | 5.36355 |
|  |  | LEU378 | A | 4.52511 |
|  |  | ILE722 | A | 4.60832 |
|  |  | VAL773 | A | 3.95789 |
|  |  | PRO294 | PA | 4.515 |
|  |  | ALA724 | PA | 4.34429 |
| OMP22 | -7.4 | SER382 | H | 2.72492 |
|  |  | VAL723 | C | 2.98738 |
|  |  | ALA374 | C | 2.95159 |
|  |  | THR377 | PS | 3.72446 |
|  |  | THR3 | PS | 3.51038 |
|  |  | THR377; LEU378 | APS | 4.45172 |
|  |  | ALA374 | A | 3.31275 |
|  |  | ALA724 | A | 3.26678 |
|  |  | ILE741 | A | 3.14432 |
|  |  | VAL743 | A | 5.25415 |
|  |  | PRO294 | PA | 5.43579 |
|  |  | LEU378 | PA | 5.17842 |
|  |  | ALA724 | PA | 3.37752 |

Here, A = Alkyl, APS = Amide-pi stacked, C = Carbon hydrogen Bond, H = Conventional hydrogen bond, HB = Hydrogen bond, HP = Hydrophobic bond, PA = Pi-alkyl, Pa = Pi-anion, PC = Pi-cation, Pd = Pi-donor, PS = Pi-sigma, PSu = Pi-sulfur, PPS = Pi-Pi stacked, PPTSh = Pi-Pi T-shaped, X = Halogen (Fluorine) bond.

**Hydrogen bond surface**

**Non-bonding interactions**

**Superimposed view of docked conformer**

**Figure Supplementary S8. Docked conformer with the targeted protein, non-bonding interactions, and the hydrogen bond surface of OMP and its analogues**

**Table Supplementary S6. Absorption, distribution, metabolism, and toxicological properties (ADMET) studies of OMP analogues**

| Name | Absorption | | | Distribution | | | Metabolism | Toxicity | | | | |
| --- | --- | --- | --- | --- | --- | --- | --- | --- | --- | --- | --- | --- |
|  | HIA | HOB | C2P | BBB | P-GpI | P-GpS | CYP450 2C9 | hERG | Carcinogen | AOT | RAT  LD50 | Hepatotoxicity |
| OMP2 | 1.0000 | 0.7571 | 0.8362 | -0.6073 | NI(0.9356) | NS(0.5539) | NS(0.7921) | WI(0.9361) | NC(0.7919) | III | 2.2968 | 0.6375 |
| OMP4 | 1.0000 | 0.7857 | 0.8362 | -0.6073 | NI(0.9356) | NS(0.5539) | NS(0.7921) | WI(0.9361) | NC(0.7919) | III | 2.2968 | 0.6875 |
| OMP6 | 1.0000 | 0.7571 | 0.8362 | -0.6073 | NI(0.9356) | NS(0.5539) | NS(0.7921) | WI(0.9361) | NC(0.7919) | III | 2.2968 | 0.6375 |
| OMP7 | 1.0000 | 0.7857 | 0.8362 | -0.6073 | NI(0.9356) | NS(0.5539) | NS(0.7921) | WI(0.8851) | NC(0.7919) | III | 2.2968 | 0.6875 |
| OMP9 | 0.9756 | 0.8000 | 0.6304 | 0.5321 | NI(0.9438) | NS(0.5848) | NS(0.8113) | WI(0.7700) | NC(0.8422) | III | 2.4305 | 0.6125 |
| OMP10 | 0.9728 | 0.8571 | 0.6360 | -0.6173 | NI(0.9327) | NS(0.6108) | NS(0.8199) | WI(0.7622) | NC(0.8260) | III | 2.4158 | 0.7125 |
| OMP12 | 0.9929 | 0.7857 | 0.6462 | -0.5262 | NI(0.9653) | S(0.5363) | NS(0.8717) | WI(0.5207) | NC(0.7915) | III | 2.4715 | 0.5875 |
| OMP13 | 0.9842 | 0.8286 | 0.6371 | 0.5321 | NI(0.9717) | S(0.5914) | NS(0.8379) | WI(0.6319) | NC(0.8170) | III | 2.4547 | 0.6875 |
| OMP15 | 0.9870 | 0.7571 | 0.5818 | -0.7988 | NI(0.9644) | NS(0.5467) | NS(0.8457) | WI(0.9607) | NC(0.7886) | III | 2.4925 | 0.6125 |
| OMP16 | 0.9870 | 0.7571 | 0.5818 | -0.7988 | NI(0.6477) | NS(0.5467) | NS(0.8457) | WI(0.9607) | NC(0.7886) | III | 2.4925 | 0.6586 |
| OMP18 | 0.9310 | 0.7286 | -0.5176 | -0.6458 | NI(0.9539) | NS(0.5349) | NS(0.7004) | WI(0.9441) | NC(0.7953) | III | 2.4682 | 0.6500 |
| OMP19 | 0.9236 | 0.7857 | -0.5081 | -0.7538 | NI(0.9476) | NS(0.5610) | NS(0.7123) | WI(0.9418) | NC(0.7795) | III | 2.4625 | 0.6586 |
| OMP21 | 0.9219 | 0.6429 | 0.5000 | -0.6917 | NI(0.8926) | NS(0.5326) | NS(0.6295) | WI(0.9107) | NC(0.7865) | III | 2.4628 | 0.6625 |
| OMP22 | 0.9539 | 0.7286 | 0.5198 | -0.8219 | NI(0.7632) | NS(0.6037) | NS(0.7589) | WI(0.9401) | NC(0.7669) | III | 2.4761 | 0.6625 |

Here, HIA= Human intestinal absorption, HOB= Human oral bioavailability, C2P= CACO-2 permeability, BBB= Blood brain barrier, P-GpI = P-glycoprotein inhibitor, PGpS = P-glycoprotein substrate, hERG = Human ether-a-go-go Related Gene, AOT = Acute oral toxicity, RAT LD50 (mol/kg) = Rat acute toxicity, I = inhibitor, NI = Non-inhibitor, WI = Weak inhibitor, NC = non-carcinogen, NS = Non substrate

**Table Supplementary S7. PASS predicted data of OMP analogues**

| Name | Gastric  Antisecretory | Anti-  ulcerative | H+/K+-transporting  ATPase inhibitor | Anti-Helicobacter  Pylori | CYP1A2  inducer | Nephritis | Hepatitis | Stomatitis | Depression | Diarrhea |
| --- | --- | --- | --- | --- | --- | --- | --- | --- | --- | --- |
| OMP3 | 0.700 | 0.767 | 0.734 | 0.670 | 0.872 | 0.830 | 0.711 | 0.839 | 0.904 | 0.709 |
| OMP4 | 0.924 | 0.875 | 0.889 | 0.813 | 0.935 | 0.901 | 0.930 | 0.916 | 0.956 | 0.900 |
| OMP6 | 0.928 | 0.915 | 0.870 | 0.956 | 0.948 | 0.922 | 0.929 | 0.932 | 0.894 | 0.819 |
| OMP7 | 0.928 | 0.915 | 0.870 | 0.956 | 0.948 | 0.922 | 0.929 | 0.932 | 0.894 | 0.819 |
| OMP9 | 0.740 | 0.817 | 0.885 | 0.698 | 0.887 | 0.842 | 0.869 | 0.811 | 0.882 | 0.804 |
| OMP10 | 0.931 | 0.948 | 0.893 | 0.801 | 0.941 | 0.908 | 0.954 | 0.937 | 0.973 | 0.941 |
| OMP12 | 0.772 | 0.887 | 0.680 | 0.689 | 0.841 | 0.755 | 0.697 | 0.846 | 0.852 | 0.681 |
| OMP13 | 0.903 | 0.928 | 0.798 | 0.849 | 0.918 | 0.872 | 0.914 | 0.909 | 0.915 | 0.878 |
| OMP15 | 0.681 | 0.800 | 0.565 | o.678 | 0.883 | 0.841 | 0.730 | 0.878 | 0.927 | 0.693 |
| OMP16 | 0.927 | 0.903 | 0.784 | o.842 | 0.941 | 0.904 | 0.931 | 0.927 | 0.969 | 0.897 |
| OMP18 | 0.866 | 0.838 | 0.564 | o.665 | 0.845 | 0.737 | 0.612 | 0.702 | 0.674 | 0.528 |
| OMP19 | 0.971 | 0.930 | 0.709 | o.797 | 0.935 | 0.858 | 0.834 | 880 | 0.817 | 0.837 |
| OMP21 | 0.903 | 0.826 | 0.559 | o.638 | 0.707 | 0.735 | 0.626 | 0.721 | 0.698 | 0.566 |
| OMP22 | 0.927 | 0.928 | 0.759 | 0.916 | 0.974 | 0.902 | 0.925 | 0.896 | 0.884 | 0.916 |
