# Supplementary material for: Computational design and cheminformatics profiling of omeprazole derivatives for enhanced proton pump inhibition of potassium-transporting ATPase alpha chain 1
Source: PLoS One. 2025 Jun 24;20(6):e0326655. doi: 10.1371/journal.pone.0326655 (PMC12186943; doi:10.1371/journal.pone.0326655)
Supplement: Attachment — Submitted filename: renamed_e9181.pdf [file pone.0326655.s006.pdf]

Reviewer Recommendation and Comments for Manuscript Number PONE-D-25-11516

Design of omeprazole derivatives for enhanced proton pump inhibition of potassium-transporting ATPase alpha chain 1; a spectrochemical, medicinal and pharmacological study

Original Submission
Chinedum Favour Ajala, MPH

Back | Edit Review | Print | Submit Review to Editorial Office

Recommendation: Minor Revision

**Custom Review Question(s):**	**Response**
**Comments to the Author**<br><br>1. Is the manuscript technically sound, and do the data support the conclusions?<br><br>The manuscript must describe a technically sound piece of scientific research with data that supports the conclusions. Experiments must have been conducted rigorously, with appropriate controls, replication, and sample sizes. The conclusions must be drawn appropriately based on the data presented.	Yes
2. Has the statistical analysis been performed appropriately and rigorously?	Yes
3. Have the authors made all data underlying the findings in their manuscript fully available?<br><br>The PLOS Data policy requires authors to make all data underlying the findings described in their manuscript fully available without restriction, with rare exception (please refer to the Data Availability Statement in the manuscript PDF file). The data should be provided as part of the manuscript or its supporting information, or deposited to a public repository. For example, in addition to summary statistics, the data points behind means, medians and variance measures should be available. If there are restrictions on publicly sharing data—e.g. participant privacy or use of data from a third party—those must be specified.	Yes
4. Is the manuscript presented in an intelligible fashion and written in standard English?<br><br>PLOS ONE does not copyedit accepted manuscripts, so the language in submitted articles must be clear, correct, and unambiguous. Any typographical or grammatical errors should be corrected at revision, so please note any specific errors here.	Yes
5. Review Comments to the Author<br><br>Please use the space provided to explain your answers to the questions above. You may also include additional comments for the author, including concerns about dual publication, research ethics, or publication ethics. (Please upload your review as an attachment if it exceeds 20,000 characters)	Strengths:<br>- This study does a great job of providing a thorough computational analysis of omeprazole derivatives, successfully identifying three potential lead compounds: OMP3, OMP19, and OMP21.<br>- By using advanced computational techniques like DFT, TD-DFT, and MD simulations, the findings gain a higher level of reliability.<br>- The ADMET and PASS predictions offer important insights into the pharmacokinetics and safety profiles of the proposed compounds.<br><br>Areas for Improvement:<br>1. Experimental Validation: While the computational work is impressive, it's important to note that in vitro and in vivo tests are necessary to truly confirm the predicted efficacy and safety of these new derivatives. The authors should clearly mention this limitation.<br>2. Clarity and Readability: Some parts of the paper, especially the sections on computational methods, use complex terminology that could be simplified for better understanding. A few minor adjustments in language would enhance readability for a broader audience.<br>3. Discussion of Clinical Relevance: The manuscript would be

	even stronger if it included a comparison of how these derivatives relate to current PPIs in terms of clinical use, potential advantages, and associated risks.<br>4. Grammar and Formatting: There are a few minor grammatical issues that should be addressed, particularly in the introduction and discussion sections.<br><br>Final Recommendation:<br>- Some minor revisions are needed to improve clarity and emphasize the importance of experimental validation.<br>- Overall, the manuscript is well done and ready for publication after addressing the suggested changes.
6. PLOS authors have the option to publish the peer review history of their article (what does this mean?). If published, this will include your full peer review and any attached files.<br><br>If you choose "no", your identity will remain anonymous but your review may still be made public.<br><br>**Do you want your identity to be public for this peer review?** For information about this choice, including consent withdrawal, please see our Privacy Policy.	No
**Confidential to Editor**<br><br>1. Do you have any potential or perceived competing interests that may influence your review? Please review our Competing Interests policy and declare any potential interests that you feel the Editor should be aware of when considering your review. If you have no competing interests, please write "I have no competing interests."	I have no competing interests.
2. Did you receive any assistance in preparing this review (e.g. from a post-doc or graduate student)? If yes, please include their name below.	No.
3. If accepted, do you think this submission should be highlighted on the PLOS ONE website? PLOS ONE does not evaluate manuscripts based on perceived significance or readership. We aim to provide tools for readers to filter and evaluate our publications. (optional)	Yes, on a more specific subject area page (e.g. Biochemistry, Atmospheric Science)
**Do you want to get recognition for this review on a Web of Science researcher profile?**<br><br>If you opt in, your Web of Science profile will automatically be updated to show a verified record of this review in full compliance with the journal's review policy. If you don't have a Web of Science profile, you will be prompted to create a free account.	Yes

Back | Edit Review | Print | Submit Review to Editorial Office
