# Supplementary material for: Computational design and cheminformatics profiling of omeprazole derivatives for enhanced proton pump inhibition of potassium-transporting ATPase alpha chain 1
Source: PLoS One. 2025 Jun 24;20(6):e0326655. doi: 10.1371/journal.pone.0326655 (PMC12186943; doi:10.1371/journal.pone.0326655)
Supplement: Attachment — Submitted filename: Response to reviewers.pdf [file pone.0326655.s010.pdf]

Review Comments to the Author

Reviewer #5:

1. The abstract lacks the study's objective and hypothesis; authors should incorporate this crucial information.

Response: Thank you for your response. We have incorporated the objectives and hypotheses into the abstract section of the current version of the manuscript. Also, considering the word limit, we had to summarize the objectives and hypothesis in one line and rewrite the abstract.

2. While the authors provided a rationale for their choice of manuscript title, terms like 'medicinal and spectroscopic' could be misleading here, as this manuscript doesn't include any wet lab studies. It's recommended that authors use terms like "computational" or "in silico" instead.

Response: Following your recommendation, we changed the title to "**Computational design and cheminformatics profiling of omeprazole derivatives for enhanced proton pump inhibition of potassium-transporting ATPase alpha chain 1"**

3. The authors did an excellent job evaluating the stability of the new analogues through various computational assessments. However, do these new analogues retain the potency and efficacy of the OMP? What about toxicity and off-target effects? Authors should address these questions, and if they can't, they should mention this in the limitations or future directions. Please keep in mind that a high docking score does not directly predict biological activity.

Response: In our in silico approach, we have evaluated and compared all the thermodynamic, spectrochemical and docking simulation results with the control, OMP, which is a marketed drug. To assess toxicity and off-target effects, we utilized the in silico ADMET and PASS predictions and compared them with omeprazole. We fully acknowledge the concern of the reviewer that *in vivo* and *in vitro* validation is necessary to portray the full picture of this preclinical research, which is our limitation and should be the future prospect. We have clearly mentioned this in the revised version of the conclusion, as per your recommendation.

4. Several references are outdated and should be revised, particularly those cited from the 1980s and 1990s.

Response: We agree that citing recent literature is vital, but these specific references (Refs. 43, 48, 53) represent the foundational works important to the methodological context. Subsequent research builds on but does not invalidate these foundational works. For reference 63, we undertook a comprehensive search but found no alternatives that validated our findings. We hope this clarification addresses your concerns, and appreciate your suggestions and recommendations.

Reviewer #6:

I agree that modifying the molecular structure of existing drugs is a strategic approach to enhancing their pharmacological properties, potentially leading to more effective and safer therapeutics. Structural alterations can influence a drug's electronic distribution, polarity, hydrogen bonding potential, and steric characteristics, which in turn affect its binding affinity, solubility, metabolic stability, and bioavailability. By rationally designing drug candidates with optimized molecular composition and substituent positioning, researchers can improve target specificity, minimize adverse effects, and enhance therapeutic efficacy. This study has effectively achieved its aim to develop novel therapeutic alternatives by refining the structural features of OMP to improve its potency, efficacy, and safety profile.
I however, suggest that the title of this manuscript be edited. While this study has adequately used spectrochemical, as well as medical chemistry and pharmacological analysis in the structural modification and refining of OMP, all of these analysis were done computationally therefore, I recommend that the title reflect that the analysis were done computationally.

Response: We deeply appreciate your constructive suggestion to refine the manuscript title for greater clarity. In accordance with your recommendation, we have revised the title to explicitly reflect the computational approaches conducted in this study. The updated title is now: "**Computational design and cheminformatics profiling of omeprazole derivatives for enhanced proton pump inhibition of potassium-transporting ATPase alpha chain 1**"

Also, are any of the newly designed OMP analogues novel? This should be included in the manuscript. As regards structural modification and optimization, I understand that the core structure of OMP was maintained, and structural modification was carried out only on R1, R2 and R3. There are three additional positions on the benzene ring of the benzimidazole moiety and one additional position on the pyridine moiety open for functional group modifications why didn't you explore any of those positions? This question would be answered using the docking studies and analysis of the protein binding site.

Response: To the best of our knowledge and literature research, none of the OMP analogues here have ever been studied, mentioned, or investigated before; hence, they are novel. As per your suggestion, we recognized the significance of describing them as novel and thus explicitly mentioned these analogues as 'novel' in the current revised version of the manuscript.

We appreciate the reviewer's insightful observation of our manuscript's unexplored positions on benzimidazole and pyridine moieties. The mentioned sites were also structurally modified and evaluated using thermodynamic, spectrochemical, and docking studies. However, these modifications showed no significant improvement in overall results, prompting a focus on candidates, which we have mentioned. Your consideration of our answer, rationale is much appreciated.

Other than the above recommendations, this manuscript is in good shape and congratulations for excellent research and well written manuscript.

Response: Thank you sincerely for your thoughtful feedback and generous appraisal of our work. We also appreciate your constructive suggestions to strengthen the alignment between the title, methodology, and overall refining the study.
