# Supplementary material for: Computational design and cheminformatics profiling of omeprazole derivatives for enhanced proton pump inhibition of potassium-transporting ATPase alpha chain 1
Source: PLoS One. 2025 Jun 24;20(6):e0326655. doi: 10.1371/journal.pone.0326655 (PMC12186943; doi:10.1371/journal.pone.0326655)
Supplement: Attachment — Submitted filename: Peer Review Report-.docx [file pone.0326655.s007.docx]

**Peer Review Report**

Peer Review of a journal article: Design of omeprazole derivatives for enhanced proton pump inhibition of potassium-transporting ATPase alpha chain 1; a spectrochemical, medicinal and pharmacological study.

**Idowu Peter Shileayo Adebayo.**

Thank you for the opportunity to peer-review this article titled “Design of omeprazole derivatives for enhanced proton pump inhibition of potassium-transporting ATPase alpha chain 1; a spectrochemical, medicinal and pharmacological study.” The article described three major omeprazole derivatives that were identified through molecular docking with potassium transporting enzymes to demonstrate improved pharmacological efficacy and improved side effect profiles over the conventional omeprazole. This will create an opportunity for future research into these agents for better alternatives to the current omeprazole.

**Overview**

The overall idea of this article was well explained and structured as contained in the abstract. The methods used were adequate to support their findings and give rooms for future opportunities to research around proton pump inhibitors (PPI)-medications with enhanced clinical benefits plus little or no side effects. Each section of the article such as abstract, introduction, methodology, results, and conclusion were adequately written and presented or documented in clear terms. Similarly, other sections like conflict of interest, funding, clinical trial number, data availability, consent for publication, ethical/consent for approval, acknowledgement, and other author’s competing interests were adequately addressed. However, the authors did not identify any limitation to this study and few other observations which shall be discussed under each section.

**Introduction**

This section was well captured by the authors. However, the authors should reword these words “health goods” in the opening sentence of the first paragraph under introduction to properly convey their intent and avoid ambiguity. Also, paragraph three is not consistent with the ‘reference number 18.’ Similarly, paragraphs three, four and five need to be properly cited. This statement in paragraph five needs to be restructured regarding the quoted reference “It is conceivable to develop a superior drug alternative by structurally altering chemical compounds (34–36).”

**Methodology**

The authors need to pay attention to spelling error “(2.1 Geometry Optimiation)”. The authors may need to look at line seven of the first paragraph under methodology for the quoted reference “(39-42)”, this was what the authors did; I don’t think the citation is necessary except the authors have justification for that. These citations should be addressed too: “These computations were performed based on the Parr and Pearson interpretation (43–46), and Koopmans' theorem (47)”. The authors should try and reference the equation to determine hardness, softness, and chemical potential as well as the molecular formular table.

**Results and Discussion**

The results were well documented with some literature backups. The authors should try and add the relevance of Radius of Gyration and Root Mean Square Fluctuation analysis to these three omeprazole derivatives of interest.

**Conclusion**

Good conclusion. The authors need to state if the observed properties of these omeprazole derivatives after subjecting them into those various analyses and modifications will make them to have better pharmacological and improved side effects profiles over the conventional medication as contained in the abstract section. Also, the authors may need to rewrite the statement in line fourteen “it was found that certain ones displayed superior results compared to others.”

**Recommendations**

-The authors should correct the observed spellings and other semantic errors as highlighted.

-The authors should check the highlighted comments under introduction, methodology, results/discussion, conclusion and make the necessary adjustment to the comments.

-They should add the limitation(s) to this study

I recommend that this article be accepted for publication after the relevant corrections must have been made.
