# Supplementary material for: Computational design and cheminformatics profiling of omeprazole derivatives for enhanced proton pump inhibition of potassium-transporting ATPase alpha chain 1
Source: PLoS One. 2025 Jun 24;20(6):e0326655. doi: 10.1371/journal.pone.0326655 (PMC12186943; doi:10.1371/journal.pone.0326655)
Supplement: Attachment — Submitted filename: Response to Reviewers.docx [file pone.0326655.s009.docx]

**Response to the reviewers:**

We would like to thank all the reviewers as well as editor for their insightful comments.

**Additional Editor Comments to the Author:**

The authors have mentioned “spectrochemical study” in the title whereas no content of the manuscript could be identified as spectrochemical analysis or results. Similarly, the terms medicinal and pharmacological study appear superfluous in the absence of experimental analysis. More importantly, how did the authors conduct the structural modifications to obtain the OMP derivatives? By mere drawing? I wonder how the feasibility of existence and stability of the derivatives can be ascertained in the absence of real or simulated tests. Instead, the authors could have searched for substructures of OMP in the chemical databases, and screen for analysis. With these, the feasibility of existence and stability of the identified derivatives can be ascertained for further study. These grey areas and those highlighted by the reviewers need to be seriously clarified before the submission can be reconsidered.

**Response:** We acknowledge the reviewer's concern about the title's reference to the spectrochemical study. Spectrochemical studies refer to the use of spectroscopic techniques such as FT-IR and UV-Vis spectroscopy to investigate a chemical substance's composition, structure, and properties. Our studies include FT-IR, UV-Vis spectroscopy, and analysis to examine the structural and electronic properties of the designed omeprazole derivatives. These techniques are essential in drug design to confirm the molecular structure, assess to help in reactivity and stability as well as electronic properties (e.g., HOMO-LUMO gap) and interaction with the targeted protein, and confirm the presence of modified functional groups in the side chain.

By mentioning the 'designing of OMP analogues,' we intended to signify the in silico aspect of our study, which is why we refrained from directly using the term 'computational pharmacological analysis.' Our ADMET analysis, an essential and commonly employed method in drug development, offers insights into the pharmacokinetic and toxicity characteristics of the synthesised derivatives, facilitating evaluations of drug-likeness, bioavailability, and safety.

The structural modifications of OMP derivatives were not arbitrarily drawn but designed using rational drug design principles. Functional groups were carefully inserted to improve omeprazole's pharmacokinetic and pharmacodynamic aspects while keeping its core structure. To assure feasibility and stability, we performed quantum chemistry and thermodynamic investigations to determine electronic characteristics and energetic favorability. FT-IR and UV-Vis spectroscopy confirmed functional groups and structural integrity, whereas ADMET and PASS predictions assessed drug likeness and toxicity. Molecular docking evaluated binding interactions, while MD simulations confirmed enzyme-ligand stability under physiological settings. While substructure searches in chemical databases are one technique, our computational assessments revealed that the proposed derivatives are chemically viable and stable, confirming their potential for further investigation.

**Reviewer #1:** 
Areas for Improvement:

1. Experimental Validation: While the computational work is impressive, it's important to note that in vitro and in vivo tests are necessary to truly confirm the predicted efficacy and safety of these new derivatives. The authors should clearly mention this limitation.

**Response:** As no funding has been received for this research, our study was limited to in silico analyses, and we could not proceed with experimental validation through in vitro or in vivo studies. We added this limitation to the conclusion of the revised manuscript.

2. Clarity and Readability: Some parts of the paper, especially the sections on computational methods, use complex terminology that could be simplified for better understanding. A few minor adjustments in language would enhance readability for a broader audience.

**Response:** According to your suggestion, we simplified the complex terminologies in the revised manuscripts.
3. Discussion of Clinical Relevance: The manuscript would be even stronger if it included a comparison of how these derivatives relate to current PPIs in terms of clinical use, potential advantages, and associated risks.

**Response:** In this study, we used omeprazole (a first-generation PPI) as the control and maintained it consistently across all analyses. Following your comment, we conducted a literature review to find relevant comparative data with other marketed PPIs but didn't find information aligning directly with our study framework. To address this, we recalculated ADMET values, which showed nearly identical results for omeprazole and esomeprazole (BBB = –0.6326, HIA = +0.9968, Caco-2 = +0.8867) and slightly different values for pantoprazole (BBB = –0.5777, HIA = +0.9935, Caco-2 = +0.7262). These confirm the appropriateness of our selected control. Our primary aim was to assess how omeprazole-derived analogues perform relative to the parent compound. The thermochemical and spectrochemical studies revealed that several OMP analogues possess good reactivity, stability, ADMET and PASS values, and strong affinity to the targeted protein. Nonetheless, we recognize the value of broader comparisons and plan to include detailed evaluations with other marketed PPIs in future studies. I hope you will consider this.

4. Grammar and Formatting: There are a few minor grammatical issues that should be addressed, particularly in the introduction and discussion sections.

**Response:** We have addressed the suggested grammatical corrections in both the introduction and discussion sections. Additionally, we thoroughly reviewed and revised the entire manuscript to ensure overall grammatical accuracy.

**Reviewer #2:**

Although the manuscript makes commendable use of a computational approach, the reliance on an AlphaFold-predicted structure for the potassium-transporting ATPase alpha chain 1 (PTAAC1) would benefit from explicit mention of the model’s confidence metrics. Stating the predicted local distance difference test (pLDDT) score or any reference to existing crystallographic data would strengthen confidence in docking results.

**Response:** We have incorporated the pLDDT values in the manuscript, which were> 90, signifying a very high level of model confidence.

The docking scores of certain omeprazole analogs are indeed superior to the original omeprazole. However, readers would welcome a brief comparison to known commercial PPIs such as pantoprazole or esomeprazole. Such a comparison would situate these findings in a broader clinical context and highlight the magnitude of improvement.

**Response:** In response to your suggestion, we initially searched the literature for relevant comparative data on the docking scores of omeprazole analogues with commercial PPIs like pantoprazole and esomeprazole. Unfortunately, we could not find direct comparative studies or docking scores for these compounds in the available literature. As a result, we recalculated the docking scores for pantoprazole and esomeprazole alongside the omeprazole analogues and mentioned them in the revised manuscript. The calculated binding energies for pantoprazole, esomeprazole, and omeprazole were -7.2 kcal/mol, -7.3 kcal/mol, and -7.0 kcal/mol, respectively, in association with the targeted PTAAC1 protein. Our results revealed that certain omeprazole analogues exhibit superior docking scores compared to omeprazole and the commercially available PPIs pantoprazole and esomeprazole, suggesting potential improvements in binding affinity and therapeutic potential.

The study thoroughly reports parameters like RMSD, RMSF, and rGyr, but it does not discuss thresholds that would characterize significant differences. Offering a straightforward explanation of how these metrics demonstrate genuine stability, beyond standard random fluctuations, would clarify the practical impact of the observed numerical variations.

**Response:** Lower RMSD, RMSF, and rGyr values are typically associated with greater stability of the molecular complex. In our analysis, the values for these parameters were consistent with those of the control, indicating that the omeprazole analogues exhibit similar structural stability. This suggests their potential as effective alternatives to conventional PPIs. To clarify the relevance of these findings, we have verified that the observed variations are within acceptable thresholds, ensuring that the differences reflect true stability rather than random fluctuations. Furthermore, we have addressed the queries raised in individual sections, explaining how these metrics demonstrate genuine stability and their implications in the context of our study.

The analyses reveal that functional groups such as –OCH_3_, –OCF_3_, –NH2, and –NHCOCH3 influence the dipole moments, free energies, and HOMO-LUMO gaps. A concise discussion explaining how each group’s electron-withdrawing or -donating nature influences both reactivity and predicted pharmacological properties would strengthen the connection to medicinal chemistry principles.

**Response:** We have thoroughly reviewed the relevant literature to better understand and explain how specific functional groups such as –OCH₃, –OCF₃, –NH₂, and –NHCOCH₃—influence key electronic parameters including dipole moments, free energies, and HOMO-LUMO energy gaps. In the revised manuscript, we have incorporated a more detailed discussion highlighting how the electron-donating or -withdrawing nature of these substituents affects the compounds’ chemical reactivity and predicted pharmacological behavior. The electron-donating or withdrawing effect of functional groups has an impact on the dipole moment, free energy, and HOMO-LUMO gaps. The strong electron-withdrawing nature of fluorine leads to an increase in dipole moment in the presence of electronegative atoms or polar bonds. The trifluoromethoxy (–OCF_3_) group exhibits the largest dipole moment, followed by acetamido group (–NHCOCH_3_), which contains both a polar carbonyl and an amide group. Through the donation of their lone pairs and the influence of resonance, the methoxy (–OCH_3_) and amino (–NH_2_) groups contribute to an increase in the dipole moment. Regarding free energy, electron-donating groups (–OCH_3_, –NH_2_) contribute to the stabilization of the molecule, leading to a decrease in free energy; in contrast, electron-withdrawing groups (–OCF_3_, –NHCOCH_2_) result in the destabilization of the molecule, thereby increasing free energy. About the HOMO-LUMO gap, the presence of electron-donating groups pushes electro into the system, therefore raising the HOMO level, decreasing the gap, and increasing molecular reactivity, while electron-withdrawing groups increase the gap by pulling electrons from the system, stabilizing the molecule and reducing its reactivity. While –OCF_3_ and –NHCOCH_3_ lead to an expansion of the HOMO-LUMO gap, thereby enhancing stability, –OCH_3_ and –NH_2_ contribute to an overall increase in reactivity. These medicinal chemistry-based modifications maximize pharmacological effects and reactivity for stability and drug efficacy. We clarified these problems in the revised work to strengthen links between medication design and other aspects.

The work relies on in silico methods, yet the text does not elaborate on the next steps in experimental verification. Explaining how these analogs might be further validated, whether through biochemical assays, cell culture studies, or animal models, would highlight translational relevance and guide future researchers toward practical implementation of the findings.

**Response:** We appreciate your suggestion to elaborate on the next steps for experimental validation of our findings. However, the lack of financing for this work is a significant challenge for us in carrying out experimental validations. We would like to request you to consider this limitation.

**Reviewer #3:** 
Overview

The overall idea of this article was well explained and structured as contained in the abstract. The methods used were adequate to support their findings and give rooms for future opportunities to research around proton pump inhibitors (PPI)-medications with enhanced clinical benefits plus little or no side effects. Each section of the article such as abstract, introduction, methodology, results, and conclusion were adequately written and presented or documented in clear terms. Similarly, other sections like conflict of interest, funding, clinical trial number, data availability, consent for publication, ethical/consent for approval, acknowledgement, and other author’s competing interests were adequately addressed. However, the authors did not identify any limitation to this study and few other observations which shall be discussed under each section.

**Response:** We have addressed limitations in the conclusion part of the revised version.

Introduction
This section was well captured by the authors. However, the authors should reword these words “health goods” in the opening sentence of the first paragraph under introduction to properly convey their intent and avoid ambiguity. Also, paragraph three is not consistent with the ‘reference number 18.’ Similarly, paragraphs three, four and five need to be properly cited. This statement in paragraph five needs to be restructured regarding the quoted reference “It is conceivable to develop a superior drug alternative by structurally altering chemical compounds (34–36).”

**Response:** All recommendations were carefully reviewed and incorporated into the revised manuscript to ensure clarity and improve the quality of work.

Methodology
The authors need to pay attention to spelling error “(2.1 Geometry Optimiation)”. The authors may need to look at line seven of the first paragraph under methodology for the quoted reference “(39-42)”, this was what the authors did; I don’t think the citation is necessary except the authors have justification for that. These citations should be addressed too: “These computations were performed based on the Parr and Pearson interpretation (43–46), and Koopmans' theorem (47)”. The authors should try and reference the equation to determine hardness, softness, and chemical potential as well as the molecular formular table.

**Response:** We’ve thoughtfully revised the manuscript based on your valuable suggestions.

Results and Discussion

The results were well documented with some literature backups. The authors should try and add the relevance of Radius of Gyration and Root Mean Square Fluctuation analysis to these three omeprazole derivatives of interest.

**Response:** We incorporated the significance of the Radius of Gyration and Root Mean Square Fluctuation analysis in the revised manuscript.

Conclusion
Good conclusion. The authors need to state if the observed properties of these omeprazole derivatives after subjecting them into those various analyses and modifications will make them to have better pharmacological and improved side effects profiles over the conventional medication as contained in the abstract section. Also, the authors may need to rewrite the statement in line fourteen “it was found that certain ones displayed superior results compared to others.”

**Response:** In the conclusion section, we have already addressed the pharmacological benefits and improved effects over conventional medication.  Additionally, we have revised the line as per your remarks.

**Reviewer #4:**

Please make the font size in figures readable. I am unable to read the details in the figures especially 9 and 11.Please explain the figures in legends. Why 100 ns simulations were recorded?

**Response:** We regenerated Figures 9 and 10 with high resolution in the result section and explained the figures in legends.

Based on evidence from the literature, 100 ns molecular dynamics (MD) simulations are commonly used and generally considered reliable enough to provide meaningful insights into molecular interactions and stability. However, running longer simulations would undoubtedly provide more robust insights. Due to limited access to high-resolution computational facilities, we conducted simulations for 100 only. We sincerely hope you’ll take this into consideration.

How can we test OMP3 and OMP21 as potential for next generation PPIs and how can we say that after computational modeling these PPIs have no side effects or less side effects?

**Response:** We investigated omeprazole and its analogues for chemical reactivity, stability, binding potential, molecular dynamics, and other pertinent properties. For further extension, advanced molecular dynamics simulations can help improve computational predictions, while structural and biophysical investigations utilising atomic resolution techniques (such as X-ray crystallography, cryo-EM, and NMR spectroscopy) can validate OMP3 and OMP21 as next-generation PPI inhibitors and confirm binding at atomic resolution. Pharmacokinetic (PK), pharmacodynamic (PD), and toxicity studies will describe drug stability, metabolism, and safety, while in vivo validation using animal models will assess efficacy. These methods will offer thorough proof of their possible therapeutic advantages.

We also evaluated each analogue's ADME (Absorption, Distribution, Metabolism, Excretion) and toxicity features and compared the results to the control's. According to the ADMET study, the derivatives had fewer adverse effects than the control. We were unable to synthesis the compounds and perform in vitro or in vivo validation at this time due to financial constraints.  Nonetheless, we acknowledge the significance of these steps in fully confirming the safety and effectiveness of the drugs.  As funding becomes available, we want to continue these projects, which need a significant investment of time and money. We appreciate your understanding, given the current limitations.
